# Community perceptions of universal health coverage in eight districts of the Northern and Volta regions of Ghana

**DOI:** 10.1080/16549716.2019.1705460

**Published:** 2020-02-03

**Authors:** Kalifa J. Wright, Adriana Biney, Mawuli Kushitor, John Koku Awoonor-Williams, Ayaga A. Bawah, James F. Phillips

**Affiliations:** a Heilbrunn Department of Population and Family Health, Mailman School of Public Health, Columbia University, New York, NY, USA; b Regional Institute for Population Studies (RIPS), University of Ghana, Legon, Ghana; c Policy Planning Monitoring and Evaluation Division, Ghana Health Service, Accra, Ghana; d Mailman School of Public Health, Columbia University, New York, NY, USA

**Keywords:** Ghana, community-based primary health care, qualitative appraisal, scaling-up, universal health coverage, health systems research, quality of care

## Abstract

**Background**: Ever since Ghana embraced the 1978 Alma-Ata Declaration, it has consigned priority to achieving ‘Health for All.’ The Community-based Health Planning and Services (CHPS) Initiative was established to close gaps in geographic access to services and health equity. CHPS is Ghana’s flagship Universal Health Coverage (UHC) Initiative and will soon completely cover the country with community-located services.

**Objectives**: This paper aims to identify community perceptions of gaps in CHPS maternal and child health services that detract from its UHC goals and to elicit advice on how the contribution of CHPS to UHC can be improved.

**Method**: Three dimensions of access to CHPS care were investigated: geographic, social, and financial. Focus group data were collected in 40 sessions conducted in eight communities located in two districts each of the Northern and Volta Regions. Groups were comprised of 327 participants representing four types of potential clientele: mothers and fathers of children under 5, young men and young women ages 15–24.

**Results**: Posting trained primary health-care nurses to community locations as a means of improving primary health-care access is emphatically supported by focus group participants, even in localities where CHPS is not yet functioning. Despite this consensus, comments on CHPS activities suggest that CHPS services are often compromised by cultural, financial, and familial constraints to women’s health-seeking autonomy and by programmatic lapses constrain implementation of key components of care. Respondents seek improvements in the quality of care, community engagement activities, expansion of the range of services to include emergency referral services, and enhancement of clinical health insurance coverage to include preventive health services.

**Conclusion**: Improving geographic and financial access to CHPS facilities is essential to UHC, but responding to community need for improved outreach, and service quality is equivalently critical to achieving this goal.

## Background

Ever since the 1978 Alma-Ata Declaration, Ghana has embraced achieving ‘Health for All’ as a national policy priority. Important gains have been registered in the Millennium Development Goal era – maternal mortality ratios declined from 760 to 319 deaths per 100,000 live births and under-five mortality rates fell from 108 to 60 per 1,000 live births [1,2]. Nonetheless, progress fell short of Millennium Development Goals 4 and 5 [1,2]. Moreover, pronounced regional health equity problems persist [3]. Mortality from preventable causes such as postpartum haemorrhage, hypertensive disorders, abortion and sepsis remain as core problems [4,5], as well as childhood infectious diseases, such as malaria, acute respiratory diseases, and diarrheal diseases [6]. These causes are amplified by pervasive poverty [7–10] and social correlates, such as low educational attainment [11] and constraints to the health-seeking autonomy of women [12]. Such circumstances attest to the need for implementation research on ways to address challenges in the provision of maternal and child care that aligns with the Universal Health Coverage (HC) agenda.

This paper responds to this evidence gap by analysing grassroots perceptions of Ghana’s flagship initiative for achieving UHC -the Community-based Health Planning and Services (CHPS) Initiative. Grounded in 25 years of implementation research [13], CHPS aims to bring primary health care to every rural community using organizing strategies originally developed and tested by a Navrongo Health Research Centre plausibility trial. Conducted from 1996 to 2003 [14] and adopted as national policy in 1999, when preliminary trial results were promising, CHPS has functioned as the core Ministry of Health (MOH) ‘Health for All’ strategy since its implementation commenced in 2000 [15,16], and later expanded to achieve UHC goals.

**Figure 1.**
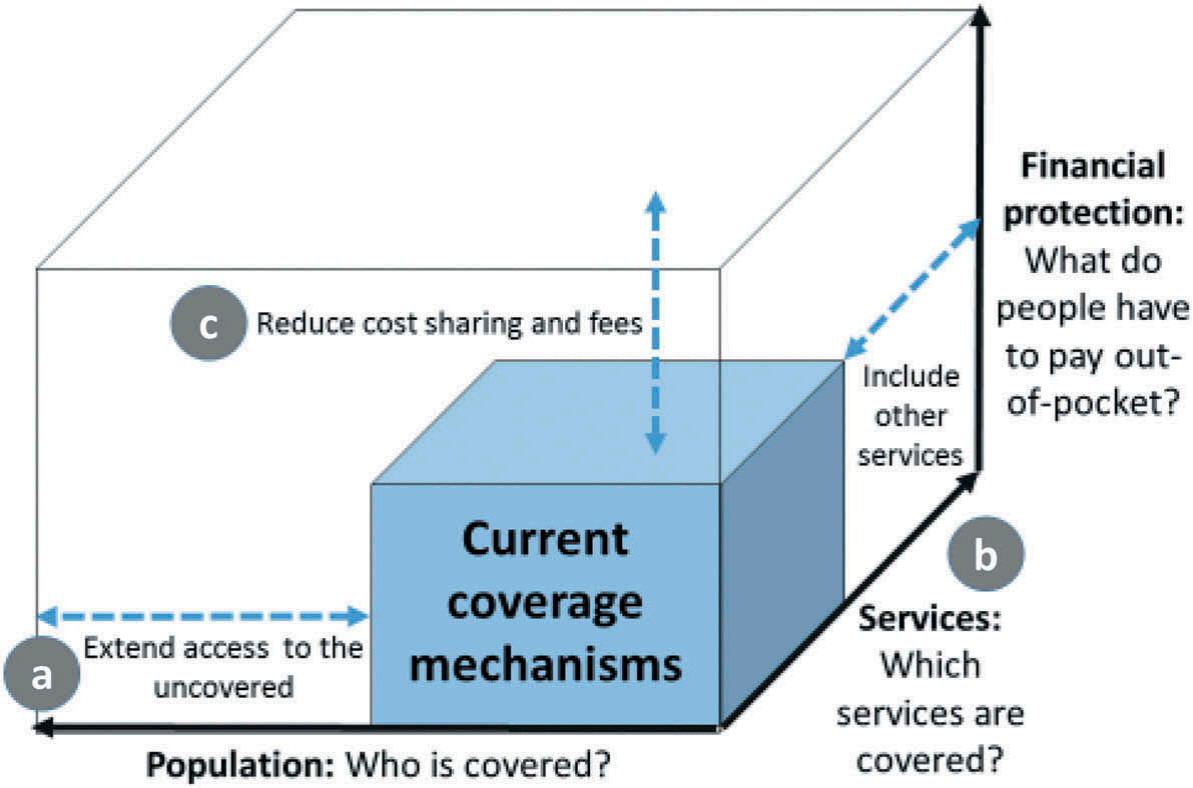
The WHO UHC cube: towards universal coverage

The World Health Organization (WHO) has summarized its commitment to UHC with the ‘UHC Cube’ diagram that appears in the 2010 World Health Report (Figure 1) [17]. These key components of UHC are reflected in the aims of CHPS. Although resource and organizational constraints slowed expansion of CHPS coverage over its first decade of operation [18], CHPS has remained central to the UHC goal of making primary health-care accessible. Trained nurses, termed ‘Community Health Officers’ (CHO), provide access to primary health care at the community level (Figure 1(a)). Improving the range and quality of CHPS services are central themes of CHPS implementation policy (Figure 1(b)). CHO provide child curative and preventive health services, antenatal care (ANC), promotion of skilled delivery supervision, post-natal care (PNC), and family planning. The development of health posts where CHO live and provide care is critical to CHPS implementation progress [19]. Once a health post is available, CHO clinical services can be conducted in conjunction with immunizations, health promotion, weekly outreach clinics, and continuous household visits [20].

A National Health Insurance Scheme (NHIS) has been launched with the goal of reducing the financial burden of seeking primary care. CHPS aims to prevent complications arising from delayed care, while also improving efficiency, effectiveness and affordability of a wide range of health services by providing care at convenient CHPS community locations, thereby augmenting the financial access goals of the NHIS [21] (Figure 1(c)).

CHPS contributes to UHC goals if operations are fully functioning [22]. Women residing in CHPS covered communities are four times more likely to receive a complete regimen of ANC and five times more likely to receive PNC compared to non-CHPS areas [23]. Health equity and childhood survival are improved in localities where CHPS is functioning [10]. Yet, despite this evidence of potential benefits, coverage is often incomplete or flawed [24–29] and NHIS implementation lapses often constrain financial access [30–32].

Monitoring evidence from the first decade of operation showed that the pace of CHPS implementation was unacceptably slow. In response, the MOH launched a study in 2008 to identify factors that explain this problem [18]. Recommendations emerging from this investigation focusing on leadership development strategies were assembled into interventions of a four district five-year experiment known as the Ghana Essential Health Interventions Programme (GEHIP) [33]. GEHIP tested means of improving the management of CHPS start-up activities and demonstrated ways to accelerate coverage, improve emergency referral services, and reduce mortality [34].

*The National Programme for Strengthening the Implementation of the Community-based Health Planning and Services (CHPS) Initiative* (CHPS+) was launched by the Ghana Health Service (GHS) in 2016 [35] to develop strategies for replicating and testing the impact of GEHIP in other regions of Ghana. A baseline CHPS+ qualitative systems appraisal was conducted to elucidate perceptions of CHPS in the Northern and Volta regions of Ghana. Within these regions, CHPS+ has created four System Learning Districts (SLD) (Nkwanta South and Central Tongu in the Volta Region, Gushiegu and Kumbungu in the Northern Region) which function as centres of excellence for disseminating GEHIP systems development strategies [36]. This paper revisits the qualitative research strategy that was used to develop and refine CHPS [26,37–39].

## Methods

This study is a component of the application of the WHO paradigm for evidence-driven scaling up that is known as the ‘Strategic Approach’ for assessing community perceptions of problems to be solved by implementation research [40–42]. Phase 1 in this paradigm is comprised of qualitative formative research that identifies problems and clarifies interventions with thematic analysis. Results identify possible operational improvements that can be subsequently put to trial. Based on the outcome of Phase 2 experimentation, replication studies and scaling up of systems reform follow [21,43]. This study was designed to clarify CHPS reform themes for trial in the CHPS+ initiative [35].

The regional coverage of CHPS in the Volta and Northern Regions is typical of national coverage (Table 1), but health and survival indicators are indicative of greater levels of adversity that the national mortality rates (Table 1). In the Volta Region, the prevailing maternal mortality ratio is over double the national average. The Volta Region is poorer than the national standard, and the Northern Region is poorer still [44]. Women with no educational attainment comprise 28.3% of the Volta Region population versus 58.9% of all women in the Northern Region. The pattern is similar for men (15.3% versus 44.3%) in the Volta and Northern Regions, respectively, [44].

**Table 1.** Estimates of key indicators of CHPS service coverage by study regions

Population characteristic	Study regions		Ghana
	Volta region	Northern region	
Projected 2016 populations	2,434,212	2,858,793	28,308,301
Demarcated CHPS zones	649	430	6,548
Functional Zones	408	291	4,400
Percent of demarcated zones that are functional	62.9	67.7	67.2
Maternal Mortality Ratio^a^	706	531	319
Skilled Delivery (%)	66.3	36.4	73.7
Neonatal Death Rates^b^	30	24	29
Under Five Mortality Rate^c^	61	111	60
Contraceptive Prevalence Rate^d^	29.5	10.8	77.2
C-section Rate^e^	8.8	2.7	12.8

^a^Deaths to women during pregnancy and 42 days following delivery per 100,000 live births (2010–2015).

^b^Rates calculated as the number of deaths within 28 days of delivery per 1,000 live births.

^c^Rates calculated as the number of deaths between birth and 5 years of age per 1,000 live births.

^d^Percent of surgical deliveries.

^e^Percent of women aged 15–49 who are currently using any modern method of contraception.

Sources [5,38,47,48].

These regional characteristics are reflected in the profile of focus group participants (Table 2). In the Northern Region, over 40% of the participants lacked any educational attainment (Kumbungu 41%; Gushiegu 44%). Study participants in Nkwanta South, a northern district of the Volta Region, had somewhat lower levels of educational attainment than Kumbungu or Gushiegu, with 61% lacking any education, 15% with primary education and only 11% with secondary education. Somewhat higher levels of educational attainment were evident in Central Tongu, where a majority of participants had middle-school educational attainment (56%) followed by primary (17%) and secondary education (16%). Thus, Northern Region residents, and study participants from that region, are more socio-economically disadvantaged than Volta Region residents.

**Table 2.** Numbers of focus group participants by SLD, type of participant and type of background characteristics of participants

Type of focus group participant	Northern region SLD		Volta region SLD	
	Gushiegu	Kumbungu	Nkwanta South	Central Tongu
Fathers	16	16	18	13
Mothers	16	16	21	15
Young boys	16	16	16	15
Young girls	14	16	14	16
Leaders	17	19	21	16
Total	115	116	122	97
**Focus group participant characteristics:**				
Educational attainment				
No education	44%	41%	61%	7%
Some education	56%	59%	39%	93%
Household religion:				
Christian	0%	0%	46%	91%
Muslim	95%	100%	3%	0%
Animist	1%	0%	27%	9%
Non-response/no religion	4%	0%	24%	0%
Total	100%	100%	100%	100%

From April to May 2017, CHPS frontline workers were convened by District Health Management Teams (DHMT), informed about study plans, and requested to mobilize participants for group discussions. Sessions were conducted by 10 male and 10 female interviewers, all of whom were hired for the study and trained in focus group methods by social scientists affiliated with the University of Ghana Regional Institute for Population Studies (RIPS) and the University for Development Studies (UDS). The CHPS+ qualitative systems appraisal consisted of 57 FGDs. However, 17 sessions for GHS district staff members, CHOs, and volunteers were excluded from the analysis, yielding 40 sessions comprised of community participants that were convened across eight communities involving 327 participants separated for mothers and fathers of children under 5, young men and young women aged 15 to 24. One session included participation of an adolescent girl who was later discovered to be 13 years old. Although panels also involved leaders comprised of elders, opinion leaders, and chiefs (Table 2), analysis of their perspectives on CHPS appears elsewhere [28], as this investigation focuses on the views of actual or potential clientele of the programme.

Sessions were conducted in communities within the four SLDs, half of which were purposefully selected among communities with functional CHPS operations while half were conducted among communities lacking functional CHPS. Discussions were conducted in local languages (Ewe, Dagbani, Likpakpaln and Twi) and subsequently translated and transcribed into English by experts in these languages. Male interviewers conducted sessions with adolescent boys, fathers and elders, while female interviewers conducted sessions involving women and adolescent girls. Interview durations ranged from 50 to 140 minutes.

Interviewers instructed participants to reflect on their experiences with CHPS, perception of the programme, and the general system of healthcare serving their locality. Participants were asked to discuss their health-seeking behaviour, delivery preferences, views on mortality risks, and family planning services. Comparisons across discussion sessions were intended to elicit a depiction of the intersection of socio-cultural norms with actual CHPS health-care experiences, providing an ‘open system’ appraisal of the adequacy of CHPS embeddedness in the local social context [19].

Each session was planned to involve eight participants. However, the size of groups varied depending on regional availability and staff allocation – yielding a total of 327 participants. The average ages of participants were 33 years for mothers, 35 years for fathers, 18 years for young girls, 20 years for young boys, and 60 years for community leaders and elders. As Table 2 shows, Gushiegu and Kumbungu are mainly Muslim communities, whereas Central Tongu communities are predominantly Christian with a minority who are traditionalists (7%). Nkwanta South communities were also predominantly Christian (46%) although a significant minority were traditionalists (27%) or participants reporting no religion (24%).

FGD guidelines invited open expressions of viewpoints on the CHPS programme by eliciting comment on the location of CHPS services and, for women, their preferred location for delivery services. Participants were encouraged to discuss their views on the causes of morbidity and mortality in their community and the role of CHPS in providing needed care. Operational features of CHPS were also discussed: service content, costs, perceptions of CHPS health-care utility and quality, the adequacy of its range of services, appropriateness attitudes and capabilities of CHPS staff, and constraints, if any, to accessing CHPS services or complying with clinical interventions or prescriptions. For each topic under discussion, participants were invited to discuss both positive and negative perceptions of the programme. For problems that were noted, participants were invited to discuss their recommendations for change.

To facilitate systematic understanding of discussion contents, analyses of 40 community transcripts utilized the qualitative analysis software Atlas.ti version 7 [45,46]. Major themes were extracted by three coders using a coding frame initially developed by deploying all coders to a common transcript, and then applying this frame as a template for coding the remainder of the transcripts.

## Results

**Figure 2.**
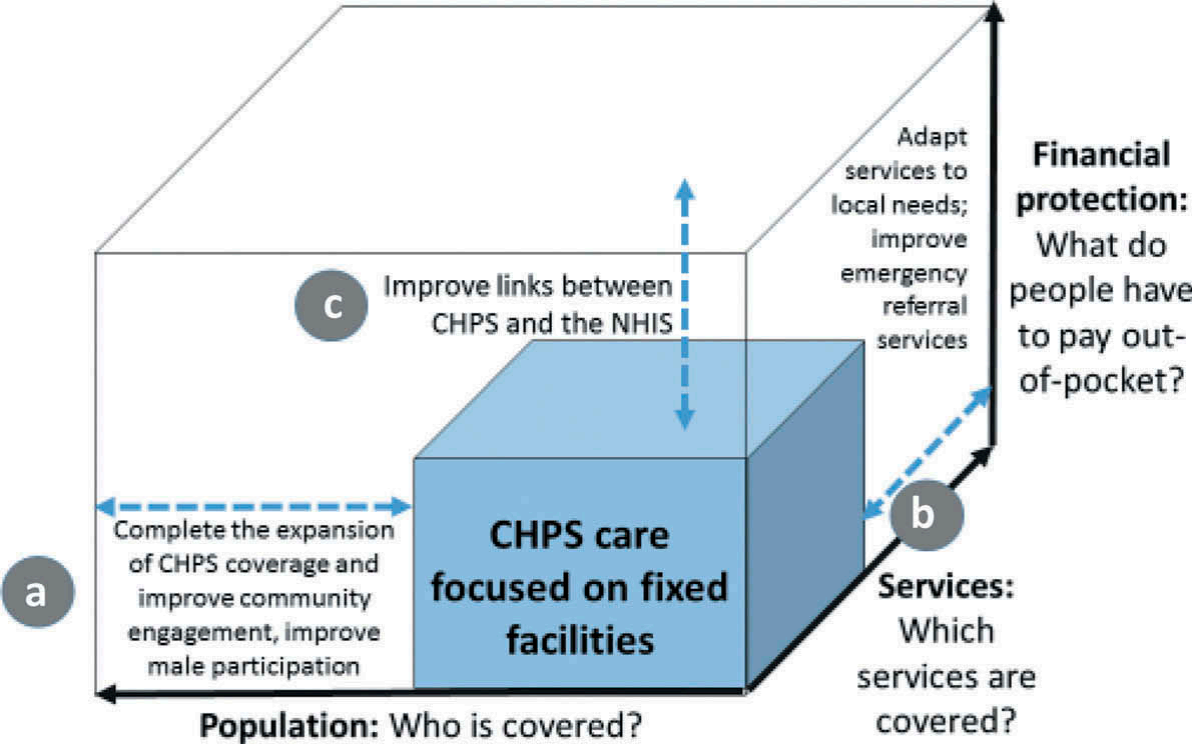
Community perceptions of improving the contribution of CHPS to achieve UHC

Thematic products of all 40 sessions covered topics that fall into all three dimensions of Figure 1 UHC cube, either as expressions of community endorsement of CHPS or discussion of programme deficiencies that merit attention and reform. Figure 2 recasts the UHC cube in terms of prominent discussant recommendations for CHPS improvement:

### The population dimension of UHC: who is covered?

All sessions yielded dialogue about healthcare access, causes of maternal and child mortality, reasons for use or non-use of services, and perceived actions and methods that CHPS could undertake to improve health-care access.

In communities where CHPS is functional, participants were generally positive about the quality and benefits of its services. Participants believed that CHPS improves financial and geographical access to health care and improved health outcomes for mothers and their children by enhancing primary care availability. Discussants acknowledged the effect of health education on expanding family planning usage, as well as improved patronage of ANC services. CHPS was described as a source of ‘strength’ that fosters community development and status.

As a matter of truth, the CHPS compound is symbolic as God is in heaven because we have suffered in the past a lot. In that, we have lost lots of lives due to the unavailability of a health facility in this wide area. There weren’t vehicles around and we had to walk very long distances before reaching places that we could get a vehicle to take us to the hospital at Adidome or Sogakope. By the time we get to the hospital, harm would have been caused … So the CHPS compound here has brought us lots of joy. It is a really good thing for us. It has come to save lives.- Community Leader (Functional CHPS community)

Participants also acknowledged the capacity for CHPS to foster regular attendance of clinics for health check-ups and ANC. Some participants expressed the view that CHPS nurses serve as role models for young girls, and provide services that reduce teenage pregnancies and delays in accessing care. These, and other positive perceptions of CHPS, have enhanced the credibility of messages rendered by workers. Although some community members were dependent on CHPS for home-based maternity services, messages were effective in communicating the risks associated with home delivery. For example, when asked about delivery preferences, a woman commented:

… I want to be healthy after giving birth. It once happened that I was pregnant and that time we had no hospital in the community. I was sick and delayed in delivering, by the time we got to the Tamale West Hospital the baby was dead in my womb. At that time if I had received early attention, I am sure I would not have lost my child. – Mother (Functional CHPS community)

This sentiment recurred in all discussions. Participants acknowledged that CHPS was, in the words of a mother, the ‘strongest weapon for our health.’ Reduced mortality was a particularly important theme:

I think we said earlier that, that was before the CHPS came here it [mortality] was happening and women and children die during child birth in the community but not now. Ever since we got the CHPS, we have not experienced such at all in this community. – Father (Functional CHPS community)

Participants from localities that lacked functioning services also believed that CHPS contributes to maternal and child health. Support for implementation is prominent even where direct access to CHPS services was lacking. However, in communities that lacked CHPS, participants noted the detrimental consequences of this circumstance:

I am not strong again because of too many births. When I wanted to give birth to my last born I suffered. I could not push. Sending me to hospital I could not sit on the motor to Nkwanta and Alokpatsa too. There was no midwife so it was not easy. Mother (Non-functional CHPS community)

### The service dimension of UHC: which services are covered?

Not all comments about CHPS services were laudatory. In all sessions, participants perceived the range of community-based, sub-district, and local hospital services to be unduly limited. Staff clinical capacity was a concern, as well as constrained outreach coverage, and sub-standard facilities or equipment [31]. Participants emphasized the need to improve access to facilities where ANC, delivery services, and post-delivery care is provided. Discussants consistently noted ways in which CHPS had failed to adapt services to community contexts. In particular, CHPS is failing to respond to the expanding burden of non-communicable diseases. Hypertension, which affects nearly a third of Ghanaian adults, in not systematically addressed by the CHO [47].

All sessions involving FGDs with women were associated with discussion of the incapability of CHPS to provide emergency referral transportation, blood transfusions or caesarean sections or other services that are vital to acute care needs. In communities where CHPS is functional, its potential programme benefits are offset by pharmaceutical unavailability, and occasional lackadaisical or ‘hot-tempered’ staff attitudes that detract from service quality.

Where CHPS is non-functional, perceptions that sub-district and hospital health workers are unwelcoming led some women to risk home delivery assisted by traditional birth attendants. District hospital care was sought only if complications arose. As other investigations have showed [48], episodes of abuse of pregnant mothers were also evident. For example:

Maybe you were one of those who never stepped in a clinic for antenatal [care], because of that you did not take any drugs that are given to pregnant women. So, when it happens that you are in labour she will also not mind you until you are suffering, and she will now ask you to go to Nkwanta. Mother. (Functional CHPS community)

When respondents were probed about the instances of maternal and child mortality related to CHPS, participants named several factors that they believed were contributing directly or indirectly to mortality, such as proximal factors related to the absence of emergency referral care and a lack of acute care capabilities at referral points. In general, responses are suggestive of a CHPS system that is isolated from a clinical care hierarchy. Respondents noted that sub-district clinics and district hospitals lacked equipment and supplies for dealing with excess blood loss, prolonged labour, and placenta retention. These deficiencies were compounded by perceived CHPS level provider negligence and clinical incompetence.

Participants expressed fatalistic views of mortality, concerns about the ineffectiveness of traditional medicine, and the inherent contradictions of simultaneously complying with the regimens of traditional and western medicine:

Another point is that some people do not fear God. If they feared Him they would value what they are carrying and make sure nothing happens to that child. There are some African beliefs that we don’t need to practice. When a woman is pregnant in places, they think some fetish priest can help the woman to deliver and sometimes it can cost the life of that woman. Father (Non-functional CHPS community)

### The financial protection dimension of UHC: what do people have to pay out of pocket?

The National Health Insurance Scheme (NHIS) is widely promoted as the health financing arm of UHC, but its reimbursement system does not function in CHPS facilities. This disconnect between CHPS and the NHIS is viewed as a critical barrier to accessing primary health care:

For the money aspect, to be honest with you, there is no money here even those who have it will tell you they don’t have it. The hospital is very important and we need it here. When it’s here and even we don’t have the money, we can fall on others for help but when it is not here it increases your expenses. Father (Non-Functional CHPS community)

As a result of policies to prevent the flow of NHIS service reimbursement revenue to CHPS facilities, all services that require prescriptions or supplies are delivered on a ‘cash and carry’ basis creating a financial burden on clients.

## Discussion

### Study limitations

Interviews conducted in local languages may have lost nuance during translation. However, all transcripts were back-translated by experts in the various languages to offset this potential problem.

Clinical functionality across CHPS service zones varies greatly, but operational details of what services the programme intends to provide may not have been fully understood by participants. Nonetheless, discussion of perceptions of CHPS were similar across locations, suggesting that a common climate of understanding of the programme prevails despite areal variation in operational functionality.

CHPS has experienced two decades of operation. It is possible that respondents believed they should express views that are consistent with the expectations of researchers or programme managers rather than valid accounts of their personal perceptions of the programme. However, the climate of candour and discussion suggests that perceptions of CHPS were authentic reflections of the program that reflected focus group participant demographics.

### Key themes

This analysis has identified three domains of participant comments, each portrayed in Figure 2 as a modification of the WHO ‘UHC Cube’ [49]. Figure 2 arrows extending from the existing primary health-care core portray features of UHC development that merit policy review. Table 3 summarizes comments, grouped according to the three Figure 2 themes. UHC policy deliberations in Ghana focus on making services as accessible, affordable, safe, effective, and comprehensive as possible by developing a unified system comprised of district hospital care, sub-district paramedical services, and CHPS (Figure 2(a)). Although GHS monitoring data show that the goal of deploying CHO to community health posts nationwide will soon be completed [50], evidence from this appraisal suggests that achieving this essential milestone will not complete the UHC agenda. Policy reform and action is needed to augment CHO posting with an extended regimen of care, including improved referral links to sub-district and hospital services (Figure 2(b)), and financial access facilitated by the reform of NHIS procedures for financing and CHPS provided care (Figure 2(c)).

**Table 3.** Perceptions of CHPS in communities with functioning services versus communities lacking resident CHPS nursing services with associated policy implications

Type of community comment	Communities with functional CHPS services	Communities lacking resident nurse provided CHPS services	Examples of possible policy implications
**Type A Comments: Population Covered**			
CHPS reduces delays in accessing care	Noted by most participants	Noted by most participants	Use as a communication theme during CHPS promotional activities.
CHPS improves infant and child health and survival	Noted by all participants	Noted by most participants	Develop CHPS links with grassroots political system
CHPS contributes to adolescent reproductive health	Noted by some participants	Noted by some participants	Develop a comprehensive adolescent health policy and strategy for CHPS
CHPS health workers serve as role models for community youth	Not mentioned	Noted by a few participants	Develop means for youth to volunteer or lead CHPS ‘durbars’
Access to care is still restricted by geographical remoteness	Not mentioned	Noted by some participants	Sustain current policies of expanding CHPS coverage
**Type B comments: service provision**			
Capacity to prevent maternal morbidity and mortality	Referral services are mentioned Facility-based delivery is promoted	Not mentioned	Review midwifery training for CHO; expand deployment of midwives to CHPS facilities
Promotion of ANC and facility delivery	Noted by some participants	Noted by some participants	Support logistics requirements of perinatal care
Health education promotes family planning adoption and spacing	Noted by some adult women participants	Noted by some adult women participants	Expand family planning outreach to men
Essential drugs are often out of stock	Noted by most participants	Noted by most participants	Urgent need for logistics and supply review and reform
Emergency referral services are lacking	Noted by some participants	Not mentioned	Strengthen operational links between service providers
Lack of confidence in technical competence of CHPS health workers	Not mentioned	Noted by few participants	Enhance technical skills of CHOs and referral systems
Negative attitudes of health workers	Noted by some participants	Noted by some participants	Launch in-service training on improving service quality and client satisfaction.
**Type C comments: financial protection**			
Offsets costs of travel for care	Noted by some participants	Noted by a few participants	Use as a communication theme during CHPS promotional activities.
Primary health care is often unaffordable	Noted by most participants	Noted by most participants	Extend NHIS reimbursement to all elements of CHPS services

CHPS is a popular concept, even among people who have yet to have its services provided in their community, as summarized in Table 3. This support for CHPS has policy implications. Ghana is a grassroots democracy where significant discretion over development investment is now vested in freely elected District Assemblies. The popularity of CHPS opens opportunities for the DHMT to build practical collaboration with development and political authorities, with prospects for expanding the co-financing of implementation operations, such as construction of health posts [51]. CHPS+ will test the implementation of this development partnership concept [35] with actions that respondents are seeking.

CHPS referral systems are not functioning, largely because operational links between CHPS and other service points in the system are not functioning. Emergency public health systems require review, with attention addressed to the feasibility of expanding the GEHIP emergency care system (Figure 2(b)). But, other limitations of service quality, lack of training, and limited services were noted by participants (Table 3). Extending CHPS coverage, without addressing service quality issues will constrain the achievement of UHC maternal and child health outcomes goals.

While completing the goal of equipping communities with health posts and CHOs remains essential to achieving UHC, access to CHPS continues to be constrained by poverty, the cost of care, and the apparent failure of the NHIS to provide timely reimbursement to providers (Figure 2(c)). CHPS workers are not entitled to insurance reimbursement for public health preventive and promotional services, leading to neglect of these components of the program. Moreover, clientele are often required to pay for essential drugs, even if they have purchased NHIS health insurance. Lack of NHIS procedural clarity and extensive delays with renewal processes lead many residents in remote communities to carry expired NHIS cards [52]. Revision of the NHIS protocol to address these problems should be pursued in conjunction with educating communities about NHIS requirements and limitations.

## Conclusion

Participants in this study who had access to the CHPS programme often attributed improvements in family health outcomes to CHPS services accessibility. Even participants who lacked access sought CHPS implementation, based on the reputation of the programme. Participants acknowledged that CHPS offers communities health education, clinical and outreach services that are otherwise unavailable. Such grassroots support attests to the necessity of continuing the process of expanding CHPS coverage as a contribution to achieving the UHC goal of equitable access to care.

Actions are also needed to address lapses in the quality of CHPS clinical and outreach services and to expand the range of modalities and care that CHPS provides to include a focus on improving referral services, given that essential emergency acute care services are beyond the capacity of CHPS personnel.

CHPS cannot succeed as a program that is solely facility-based. Improving the frequency and content of community engagement will improve awareness of the appropriate roles and capabilities of CHPS. Commentaries have acknowledged the need for UHC to extend beyond the provision of community-based care to include the active engagement of community leaders and social networks and strategies for marshalling traditions of risk-sharing and support [53,54]. Capabilities that contribute to maternal and child mortality reduction will be enhanced if strategies are put in place that combine the vibrant and robust features of Ghanaian social organization with features of the organization of primary health care that make UHC fully functional.

## Data Availability

By agreement among partners to the CHPS+ initiative, de-identified qualitative data are available to institutions seeking access by written request to the Corresponding Author of this publication. Requests should clarify the purpose of research and should specify relevant ethical review for the proposed research. Data acquired by this process are for scholarly purposes only and may not be sold or independently distributed.

## Paper context

The Ghana Health Service implemented a community-based health-care program in 2000 that is projected to complete national coverage by 2022. Achieving primary care accessibility is being pursued in conjunction with implementing a national health insurance scheme. Taken together, these programs are assumed to constitute ‘universal health coverage’ . Community perceptions of program implementation are analyzed. Results suggest that quality of care improvement and insurance system reform require action if UHC is to be realized.
